# Health information system concept in health services in the national health insurance (JKN) era in Indonesia: An environment and one health approach

**DOI:** 10.3389/fpubh.2022.952415

**Published:** 2022-10-13

**Authors:** Maria Holly Herawati, Sri Idaiani, Maryati, Fitriana, Lucitawati, Meita Veruswati, Karina Hoekstra, Al Asyary

**Affiliations:** ^1^National Research and Innovation Agency, Jakarta, Indonesia; ^2^Health Sciences College of Abdi Nusantara, Jakarta, Indonesia; ^3^Faculty of Health Science, Universitas Muhammadiyah Prof. Dr. HAMKA (UHAMKA), Jakarta, Indonesia; ^4^Institut für Informationsmanagement Bremen (IFIB), University of Bremen, Bremen, Germany; ^5^Department Environmental Health, Faculty of Public Health, Universitas Indonesia, Depok, Indonesia

**Keywords:** health information system (HIS), health service, national health insurance, public health, one health approach

## Abstract

The health information system is a component of the healthcare system. The health information system in health services in Indonesia has experienced many problems in getting support for policy making, the implementation of the industrial revolution 4.0, and national health insurance (JKN). To answer the above problems, it is necessary to make a concept of health information systems in health services that based on environment and one health perspectives. This research was part of the thematic research of the 2019 JKN National Health Facilities Survey (Rifaskes) in Indonesia. The systems approach and cross-sectional research were carried out by collecting quantitative data. A structural equation model with Lisrel 88 software was used to model the health information system. The health information system produced a concept that included the following structured input components: governance, human resources, infrastructure, types of information system (IS) (program, JKN, management), and financing; process components: funding, technical guidance, and verification and validation; and output components: open access, standards and quality, utilization, bridging, and security. The concept for strengthening the health information system prioritizes improving the output components (standards, utilization, bridging, open access, and security) in the process components (funding, verification, technical guidance) while the input components (financing, human resources, governance, IS programs, infrastructure, IS JKN, IS management).

## Introduction

Indonesia implements sustainable development in the health sector based on the principle of non-discrimination. Sustainable development begins with strengthening the system, which in this case is the health information system. The health information sys-tem can only be the basis for decision-making if the data is accurate, open, and interoperable, which was called one data in 2018 (1–6). The health information system in Indonesia faces several problems: Indonesia is currently still implementing the industrial era 4.0, while the world is already in the industrial era 6.0; information systems have not yet been merged or are not interoperable, both in the implementation of routine information systems and in implementing JKN; and time is not spent prioritizing developing technology, developing open standards, and managing security and privacy in health information systems (7–14).

In this case, the Ministry of Health is like a health organization, where this organization has service efforts in the form of hospitals, health centers, and clinics. The Ministry of Health also has administrative management at each level, from the central administration all the way to the smallest sub-district. Health service efforts with these administrations aim to make health services more accessible for communities in order to promote a healthy society (14, 15). To achieve its goals, the healthcare organization requires costs, technology, and planning as part of the identification of critical success factors. Improving the quality, accessibility, and sustainability of health services as well as increasing the availability and quality of health data or information and changes in the world in various sectors have an impact on health services in general and health information systems in particular (16).

In this article we propose an environment and one health framework for developing a high-level prospective concept of the implementation of health information systems in healthcare organizations. Based on the existing framework, we identified several critical dimensions that exist in the health information system, namely, management, financing, human resources, infrastructure, and the types of existing information systems (management information systems, program information systems, and JKN information systems) as an effort to achieve national health goals at the sub-district, district, provincial and national levels. This captures two important characteristics in the healthcare delivery process: the level of mediation and the level of internal and external collaboration.

## Materials and methods

### Ethical approval

This paper was developed based on the results of research on health information systems with Ethics Permit LB.02.01/2/KE.186/2019 (10).

### Research period and location

The study was conducted in 2019 (July–December) in 7 provinces (Aceh, West Kalimantan, Central Sulawesi, South Sulawesi, NTB, West Java, and West Papua) in 103 districts with 420 healthcare units (400 health centers and 20 hospitals) (10). The selected study's location is regarded to Indonesia's representative multi-stage clustered sampling technique.

### Research question

What is a mathematical concept for implementing the health information system in the implementation of JKN in Indonesia?

### Data collection

This paper was part of the thematic research on health information systems in Health Facility Research 2019 and used the cross-sectional method. Quantitative data collectors used questionnaires that have been tested in their work. These tested instruments were checking its validity and reliability result before it was done to collect the information in healthcare facilities. Respondents were service officers of both hospitals and health centers who handled management data, program data, and JKN data. The questionnaire's structure followed that of the system research, which included input, process, and output categories. Inputs consisted of internal (HR, governance/leadership, infrastructure, financing, types of health services, management information systems, program information systems, and JKN information systems) and external (Border Remote Areas and Islands (DTPK) fiscal capacity) topics. Processes covered funding, technical guidance, and verification. And outputs consisted of quality standards, data utilization, bridging/interoperability, open access, and security. The researchers used a generic health facility research data collection team that had been retrained for the purposes of this thematic data collection. Researchers supervised the collections twice to maintain the quality of the research.

### Data analysis

The analysis required structural equation modeling using the Lisrell method (16). The first step of our modeling was to normalize all variables, and after all variables were normal, then a good initial estimate was made of the latent component of the input with several forming variables (management, human resources, infrastructure, funding, management information systems, and program information systems, information on JKN, types of health services, health service facilities according to DTPK status, fiscal capacity status), the latent component of the process with several forming variables, and the latent component of the output with several forming variables. The input latent was an exogenous variable (X), and the process latent was an endogenous variable. For the next modeling, the process latent was an exogenous variable for the latent output, which was the final endogenous variable (Y).

## Results

**Figure 1:**
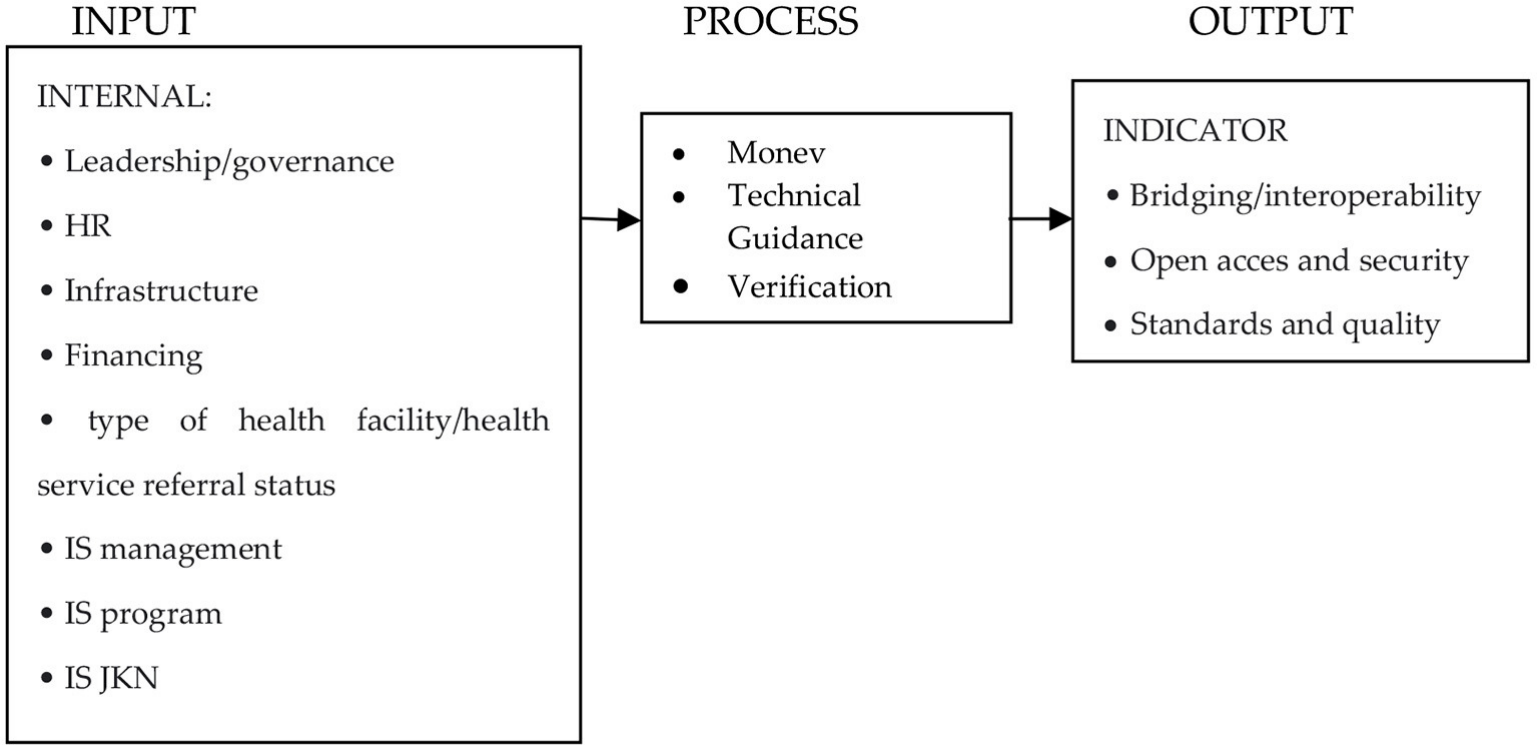
General form of structural equation modeling of the health information system in health services in the implementation of JKN in Indonesia.

The research revealed several very useful findings, which were largely missing in existing literature. Several variables formed the input component: leadership/governance, human resources, infrastructure, funding, management information systems, program information systems and JKN information systems, types of health services, health service facilities according to DTPK status, fiscal capacity status. Thus, the variables of monitoring and evaluation, technical guidance, and verification and evaluation form the components of the process. Furthermore, the standard and quality variables, data utilization, bridging and interoperability, open access and security, formed the output components (Figure 1).

### Input modeling

**Figure 2:**
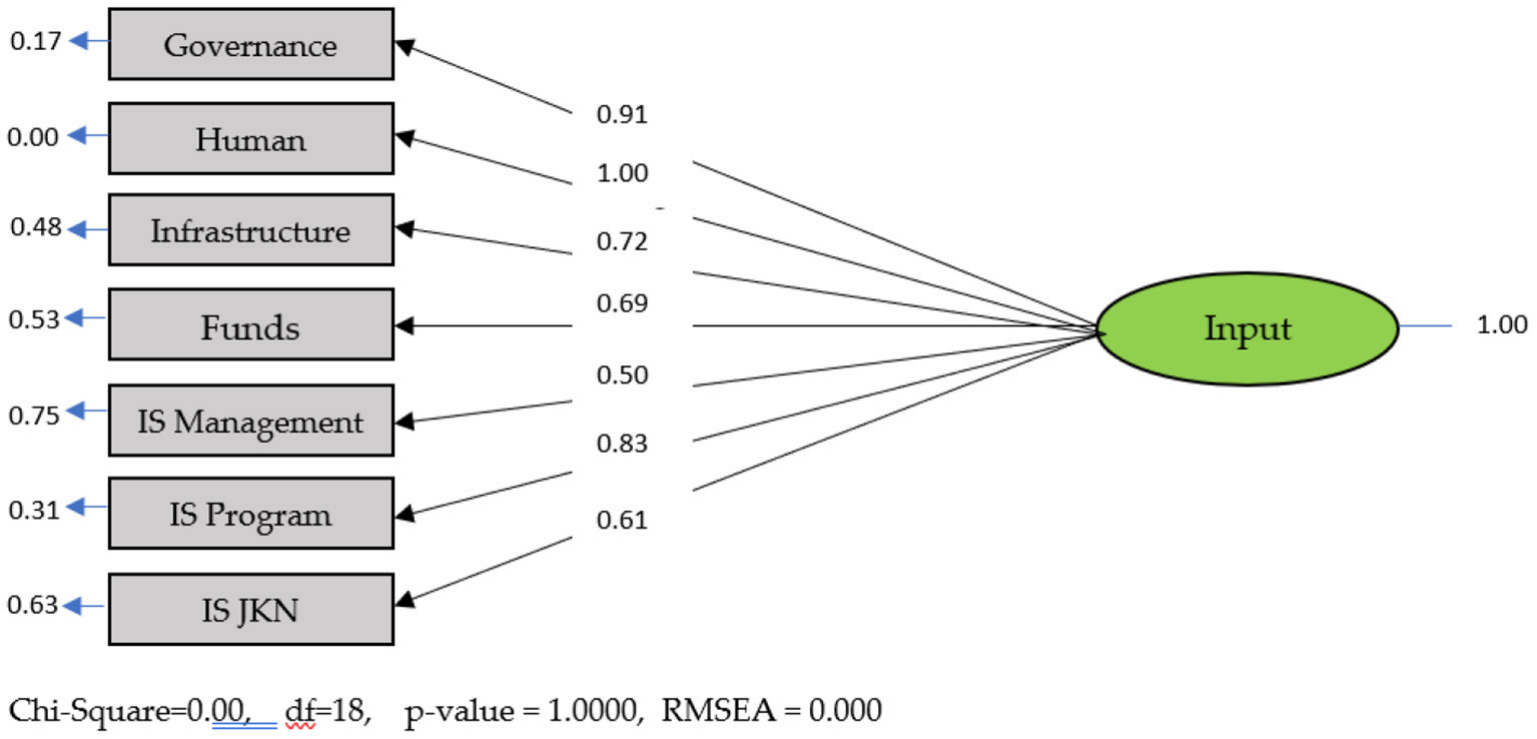
Standard input model solution.

Information system modeling is essentially input modeling with several steps, namely, making an initial estimate and making a final estimate. The final estimation results of indicators that met the requirements of good validity were governance, human resources, infrastructure, funds, IS management, IS Program, and IS JKN [absolute value of Standardized Factor Loading (SFL) > 0.50]. However, several indicators had an SFL <0.50 (DTPK, Fiscal, and Type of Health Facility) and were thus be excluded from the measurement model (Figure 2).

### Process modeling

**Figure 3:**
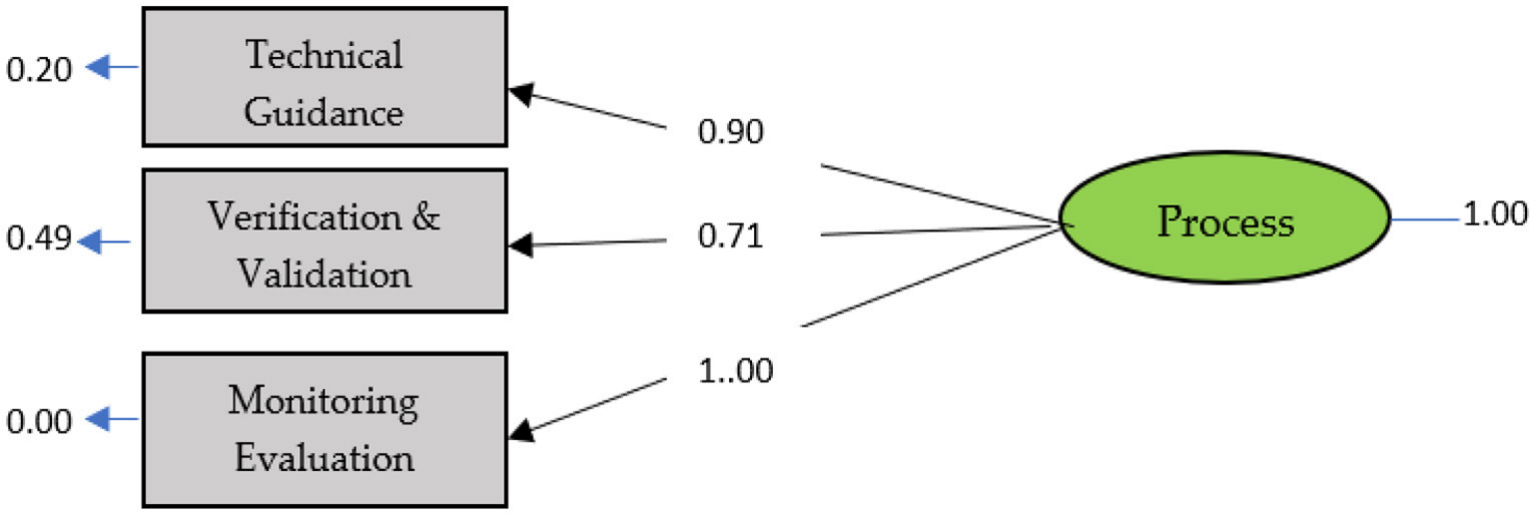
Standard process model solution.

The process modeling shows the initial estimation and final estimation of the indicators that comprised the process components that met the requirements for entering the measurement model. The three indicators that comprised the process component can be seen in the (Figure 3).

### Output modeling

Output modeling produces initial estimates of 5 constituent variables.

**Figure 4:**
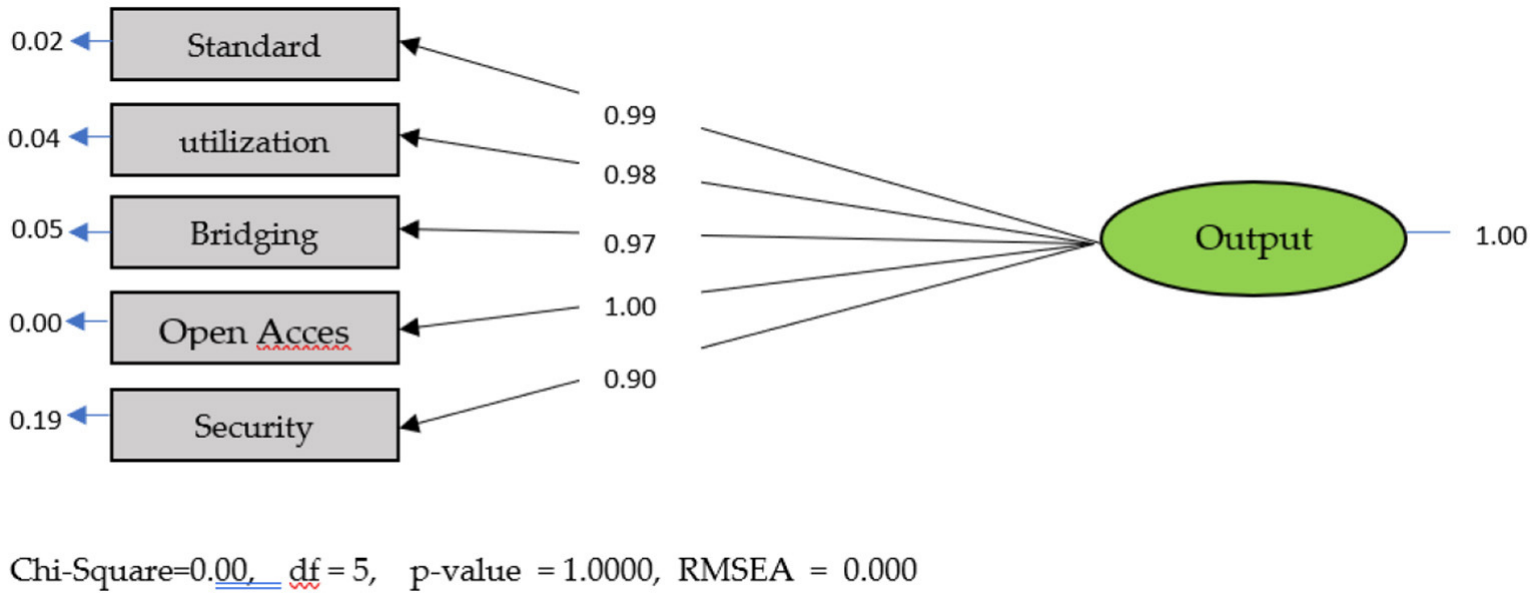
Standard output model solution.

The Path Diagram shows that the SFL of Standard, Utilization, Bridging, Open Access, Security ≥ 0.50. The five indicators are valid indicators for the output variable. The next modeling forms a full model using the inputs, processes, and outputs and considers SFL, composite reliability, and validity. The (Table 1) and (Figure 4) below show the full fit model.

**Table 1:** Results of measurement model analysis.

**Indicator variables**	**Standardized factor loading (SFL)**	**Composite reliability (CR) Variance extra (VE)**	**Conclusion**
INPUT		CR = 0,91;	Good validity
		VE = 0,59	
Governant	0,91		Good validity
Human resources	1,00		Good validity
Infrastructure	0,72		Good validity
Fund	0,69		Good validity
Management IS	0,50		Good validity
Program IS	0,83		Good validity
JKN IS	0,61		Good validity
Process		CR = 0,91;	Good reliability
		VE = 0,77	
Technical guidance	0,90		Good validity
Verification & validation	0,71		Good validity
Monitoring evaluation	1,00		Good validity
OUTPUT		CR = 0,99;	Good reliability
		VE = 0,94	
Standard	0,99		Good validity
Utilization	0,98		Good validity
Bridging	0,97		Good validity
OpenAccess	1,00		Good validity
Security	0,90		Good validity

CR, Composite Reliability; VE, Variance Extracted. CR 0.70 and VE 0.50 Good Reliability.

### Structural model analysis

**Figure 5:**
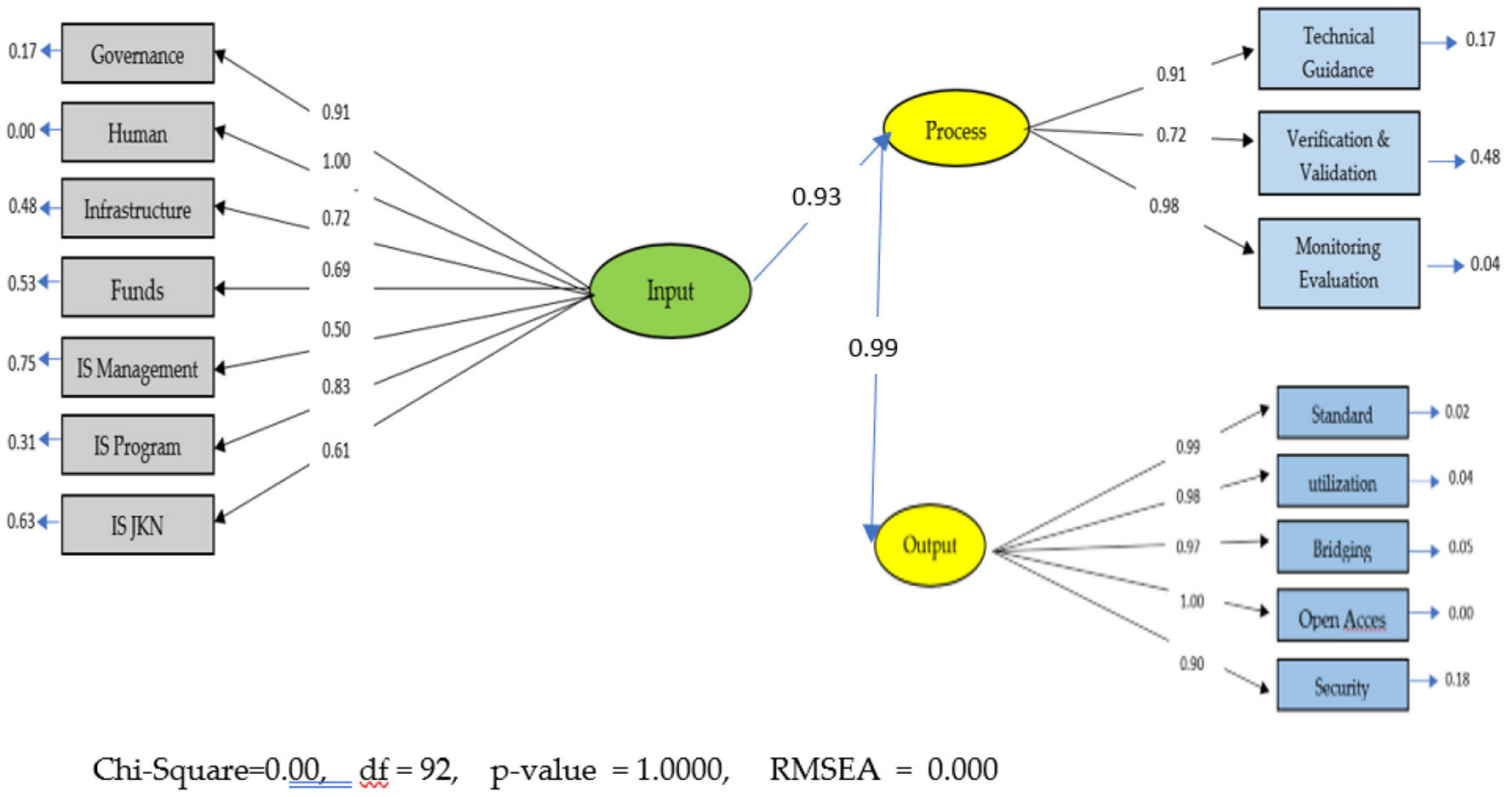
Complete model standard solution.

The (Figure 5) below illustrates the structural model and the results of the analysis. The (Table 2) below presents t values and coefficients.

**Table 2:** Results of structural model analysis.

**Path**	***t*-value**	**Coefficient**	**Conclusion**
Input → Process	21.84	0.93	Significant positive
Process → Output	41.23	0.99	Significant positive

From the Table 2 above, it can be interpreted that the results of the modeling analysis indicate that the health information system would be ready to be implemented with improvements in the outputs, then inputs, and then the processes. This means that the health information system already exists, but the output needs improvement in monitoring and evaluation and verification and validation, while the recommended output is improvement in output starting from open access, utilization, standards, bridging, and security. The input components to the process need to be improved in HR, Health Information System Governance, IS program, Infrastructure, Funds, IS JKN, and IS management.

## Discussion

The results showed that the indicators of leadership/governance, HR, infrastructure, financing, IS management, IS Program, and IS JKN comprise the input components of the health information system in this modeling. Regulations that relate to the results of this study are the 2012 National Health System (SKN, 2012) article 2 paragraph 1, which states that health information is part of health management. Furthermore, Article 3 states that in the management subsystem includes health information and regulation. In the implementation of the JKN health information system at the public health center, the health information system was constrained by the lack of internet and human resources (17). To control the implementation of information systems in an organizational system, human resources and technology are important (18–20). Likewise, an organization must prepare human resources to adapt to face problems in the implementation of the system, especially information systems (21). Improving the health information system requires 3 components: the performance of the information system, which in this paper is included as infrastructure; the support organization, which is termed in this paper as governance; and feedback from the health facilities as end-user. All three are necessary for a process to maintain quality (22).

In implementing the health information system at the health office, there were problems with human resources, supporting facilities, and commitment from the activity leaders who had not been trained to understand good data and information management (23, 24). As an analogy for this result from previous study on implementing the health information system at the Minahasa District health office, the manager of the health information system said that the most frequent obstacles were puskesmas reporting late, carrying out manual reporting, having no guidelines, lacking human resources in the field of health information systems (HIS), and lacking training, funds, facilities, and infrastructure (electricity, computers and internet) (25).

Further modeling finds that several indicators that make up the process components are technical guidance, monitoring, and evaluation as well as verification and validation. In some cases of recording and reporting systems, if there is no monitoring by village supervisors and program holders, it will usually be late. Because it is late, the head of the puskesmas often does not have time to check and dispose of it. Thus, it is signed and reported to the health office (11, 25). The results of other studies also stated that there was a need for agreement on the use of terminology standards so that the integration of various data sources can be done electronically (26).

The results of modeling the output components revealed the vital indicators of standards, utilization, bridging, open access, and security. The final modelers with the highest influence are HR, governance, program, infrastructure, funds, IS JKN, and management. In the process component, it is necessary to successively improve the health information system in monitoring and evaluation, technical guidance, verification and evaluation. Meanwhile, the output components are open access, standards, PE, the use of bridging, and security. Several studies have found that health information systems need bridging or interoperability. Too many health centers using health information systems require users to enter the same health data repeatedly, especially about the patient's social identity and clinical data, which makes the work inefficient (27). There needs to be a web-service-based bridging system development concept developed from the results of the prototype design of this health service information center that can meet user requests, such as users getting information on doctor's schedules, hospitalization, total blood stock nationally, pharmacies, reporting in the event of a disease outbreak, and reporting health agencies that have poor service (28). With the implementation of an information system with the web, the input of the health information system will be well documented in the system (18). Another study stated that the existing health information system has not run well because the recording and reporting process is still done manually, there is a lack of data security and a lack of data integration, and the information produced is not in accordance with the needs for decision-making (29). The design of the HIS at WEB-based mobile health centers overcomes the inability to provide the required information quickly, accurately, and on time manually through the information system (30).

The health information system is a means to support health services provided to the community. An effective health information system provides information support for the decision-making process at all levels, even at puskesmas or small hospitals. Not only data, but also complete, precise, accurate, and fast information can be presented with a well-organized and well-implemented health information system (16, 31). The health information system must have secure conditions (32). The results of several research projects suggest that the implementation of health information system integration will be carried out with policy support (33). Technology, which is part of the infrastructure in the input in this study, will facilitate the steps needed to achieve the output (33). In the health information system, data standardization is needed (34). Some studies argue that routine data is vital in making decisions as a basis for policy determination (34, 35). Another article mentions the role of doctors have in implementing health information systems (HR) in health services by improving the quality of health information systems as an effort to improve health services (36). Health information technology is indeed not the only effort to complete an information system, but it needs other components such as timely, complete, and accurate data as well as support from organizations such as feedback on reports and so on (37). This all will certainly affect the quality of information system data (38).

## Conclusion

The health services concept in the JKN era for strengthening the health information system that based on environment and one health approach is formulated by each components including standardized and utilized input, process, and output. These can include factors such as bridging, open access, and security. In process, the recommend strengthening components, especially funding, verification, technical guidance. It is also important to strengthen input components: funding, human resources, governance, information system programs, infrastructure, JKN information systems, and information system management.

## Data availability statement

The raw data supporting the conclusions of this article will be made available by the authors, without undue reservation.

## Ethics statement

The study was approved by the Institutional Ethics Committee of Indonesian Ministry of Health (LB.02.01/2/KE.186/2019). Written informed consent was obtained from the participants.

## Author contributions

All authors listed have made a substantial, direct, and intellectual contribution to the work and approved it for publication.

## Funding

This research was funded by the National Institute for Health Research and Development, Indonesian Ministry of Health. The publication was also funded by the Hibah PUTI UI 2022 (NKB-455/UN2.RST/HKP.05.00/2022). The funders had no role in the design of the study; in the collection, analyses, or interpretation of data; in the writing of the manuscript, or in the decision to publish the results.

## Conflict of interest

The authors declare that the research was conducted in the absence of any commercial or financial relationships that could be construed as a potential conflict of interest. The reviewer ID declared a shared affiliation with the author(s) AA to the handling editor at the time of review.

## Publisher's note

All claims expressed in this article are solely those of the authors and do not necessarily represent those of their affiliated organizations, or those of the publisher, the editors and the reviewers. Any product that may be evaluated in this article, or claim that may be made by its manufacturer, is not guaranteed or endorsed by the publisher.
